# Transcripts of wheat at a target locus on chromosome 6B associated with increased yield, leaf mass and chlorophyll index under combined drought and heat stress

**DOI:** 10.1371/journal.pone.0241966

**Published:** 2020-11-09

**Authors:** Jessica Schmidt, Melissa Garcia, Chris Brien, Priyanka Kalambettu, Trevor Garnett, Delphine Fleury, Penny J. Tricker

**Affiliations:** 1 School of Agriculture, Food and Wine, The University of Adelaide, Adelaide, South Australia, Australia; 2 The Plant Accelerator, Australian Plant Phenomics Facility, The University of Adelaide, Adelaide, South Australia, Australia; Mahatma Phule Krishi Vidyapeeth College of Agriculture, INDIA

## Abstract

Drought and heat stress constrain wheat (*Triticum aestivum* L.) yields globally. To identify putative mechanisms and candidate genes associated with combined drought and heat stress tolerance, we developed bread wheat near-isogenic lines (NILs) targeting a quantitative trait locus (QTL) on chromosome 6B which was previously associated with combined drought and heat stress tolerance in a diverse panel of wheats. Genotyping-by-sequencing was used to identify additional regions that segregated in allelic pairs between the recurrent and the introduced exotic parent, genome-wide. NILs were phenotyped in a gravimetric platform with precision irrigation and exposed to either drought or to combined drought and heat stress from three days after anthesis. An increase in grain weight in NILs carrying the exotic allele at 6B locus was associated with thicker, greener leaves, higher photosynthetic capacity and increased water use index after re-watering. RNA sequencing of developing grains at early and later stages of treatment revealed 75 genes that were differentially expressed between NILs across both treatments and timepoints. Differentially expressed genes coincided with the targeted QTL on chromosome 6B and regions of genetic segregation on chromosomes 1B and 7A. Pathway enrichment analysis showed the involvement of these genes in cell and gene regulation, metabolism of amino acids and transport of carbohydrates. The majority of these genes have not been characterized previously under drought or heat stress and they might serve as candidate genes for improved abiotic stress tolerance.

## Introduction

Climate change is a threat to future food security. Prolonged drought periods and heatwaves are predicted to become more common by the end of the century having a major impact on economically important crops such as wheat [1, 2]. Heatwaves in 2003, 2010, 2018 and 2019 broke temperature records across Europe, with 2019 one of the hottest and driest summer ever recorded [3–5]. In 2016, yield losses of between 17 and 45% of total production (corresponding to ~8 million tonnes) were recorded in France, one of the biggest wheat producers in Europe. Losses were associated with high Autumn temperatures and the compound effects of precipitation changes that are predicted to recur more frequently in the future [6]. In Australia, nine of the ten warmest years on record have occurred since 2005 with rainfall and wheat yields below average, so that the climate was both hot and dry [7, 8]. To minimize yield losses and to keep up with future food demand, the development of climate resilient wheat varieties is required.

One way to develop more resilient crops is the identification and integration of quantitative trait loci (QTL) and the underlying genes associated with abiotic stress tolerance. QTL have been identified for yield in low-yielding growth environments experiencing drought, heat or combined drought and heat stress (reviewed in [9]). However, phenotyping for grain yield on its own might not be enough to contribute significantly to cultivar improvement given the complexity of the genetic control of stress tolerance (i.e., multigenic, low heritability with high genotype by environment interactions) [10, 11]. The dissection of these QTL into their component physiological traits, which then can serve as target traits for breeding in dry and hot climates, is of similar importance. Potential key traits that have been suggested for drought and heat stress tolerance are the regulation of the water use in plants and the adaption of photosynthetic assimilation to improve radiation use efficiency [9, 12, 13].

Differentially expressed candidate genes under combined drought and heat stress have been identified in tetraploid durum wheat. Combined drought and heat stress triggered the expression of genes encoding trans-membrane proteins, as well as proteins involved in fatty acid β- oxidation [14, 15]. In bread wheat, significant genetic marker-trait associations were identified under combined drought and heat stress on group 7 chromosomes and were associated with phytoene synthase 1, integral membrane glycoproteins and a protein conferring rust resistance [16].

We developed lines (NILs) that targeted a QTL on the long arm of chromosome 6B of hexaploid bread wheat (*Triticum aestivum* L.). The QTL (*QYld*.*aww-6B*.*1*) had previously been identified in three independent studies. In semi-controlled conditions [17], the allele predominantly occurring in Asian accessions contributed to higher total grain weight per plant, single grain weight and harvest index for the heat response under drought, while decreasing screenings (% small grain weight) under drought. However, a different effect was observed in hot conditions in the field with the Asian allele reducing harvest index [18]. In controlled conditions [19], the QTL was associated with single grain weight, leaf chlorophyll content following heat stress and for the heat susceptibility index. To find candidate genes associated with the QTL, we performed a gene expression analysis of developing grains collected during early and late drought as well as with drought and heat stress during the grain-filling period. Further, by studying physiological traits such as water use and photosynthesis, we aimed to identify important tolerance mechanisms associated with drought and heat stress tolerance at this locus, which could potentially be used as target traits in crop breeding.

## Materials and methods

### Plant material

**Fig 1:**
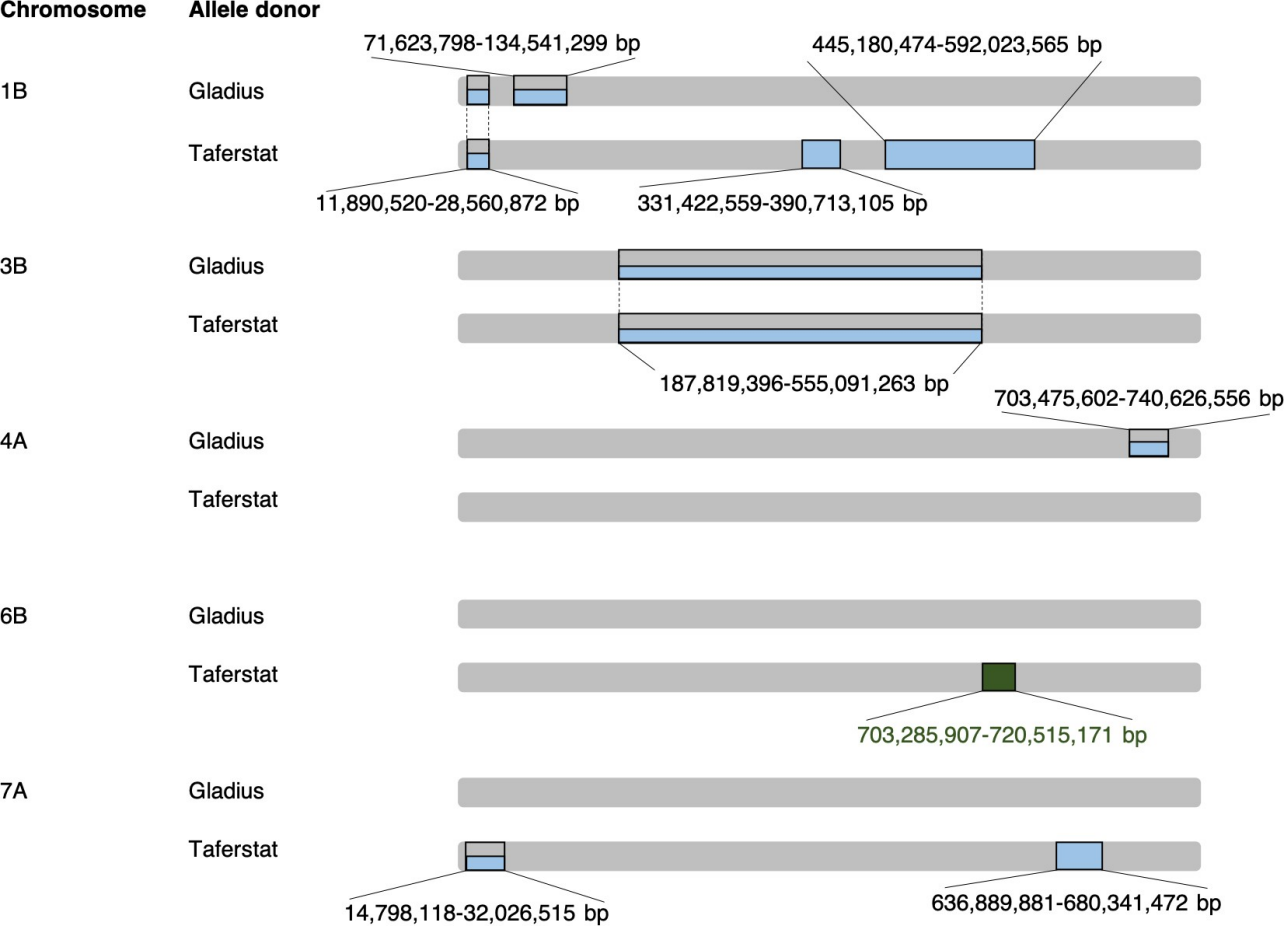
Illustration of segregating regions within near-isogenic lines at BC1F6 carrying either the allele donated by the non-exotic (Gladius) or exotic (Taferstat) parent at the target region on chromosome 6B (marked in green). Additional regions of segregation were observed on chromosomes 1B and 7A (marked in blue). Regions on chromosomes 1B, 3B, 4A and 7A, which appear to segregate between replicates, are boxed in blue-grey. NILs were homozygous for the remaining 16 chromosomes. Two BC1F6 seedlings were used per line of the Gladius/Taferstat BC1F5 NIL pair.

NILs targeting the *QYld*.*aww-6B*.*1* were developed from an existing nested association mapping population. The nested-association mapping population consisted of 73 diverse (‘exotic’) donors which were crossed with two recurrent (‘non-exotic’) Australian varieties (cvs. Gladius and Scout), back-crossed with the corresponding recurrent parent and selfed over three generations. A total of 772 recombinant inbred lines of 34 families at BC1F4 were genotyped with the SNP marker “BobWhite_c27364_296” (S1 Table). The development of molecular markers and genotyping was performed using Kompetitive Allele Specific Polymerase Chain Reaction (KASP^TM^) technology. Subsequently, the selected SNP marker was used to genotype 663 single seed descendants from heterozygous recombinant inbred lines (BC1F5), identifying pairs of NILs carrying the allele from either the non-exotic or exotic parent at the target region. Additional molecular markers developed in-house (S1 Table) revealed a NIL pair, resulting from a cross between the Australian variety ‘Gladius’ and the Algerian variety ‘Taferstat’. Seeds (BC1F6) for each line, derived from a single plant, were used for phenotyping and genotyping. DNA of two seedlings of each line was isolated using the DNeasy 96 Plant Kit (Qiagen, Germany) according to the manufacturer’s instructions, of which one sample carrying the non-exotic allele failed (S2 Table, Fig 1). DNA of two additional samples from plants carrying the non-exotic allele was isolated (as before) of which one was similar (sample ID: 22) and one different in its phenotype (sample ID: B23, reduced biomass and plant height) in comparison to the other replicates. A targeted genotyping by sequencing (tGBS) analysis based on data from the 90k SNP Illumina array was carried out by Agriculture Victoria Research (AgriBio, Australia), resulting in 9,424 markers which contained no missing values and could be mapped to a unique position within the bread wheat ‘Chinese Spring’ reference genome sequence, RefSeq v1.0 [20].

### Plant growth conditions

#### Growth chamber and glasshouse conditions

Plants were vernalized according to [21]. For vernalization, 32 seeds were initially germinated for 24 hours in Jiffypots™ at room temperature (20 ^o^C) in a reach-in chamber, followed by 4 weeks at 4–8 ^o^C with a 2-hour photoperiod (2h/22h day/night) and well-watered conditions. At the end of vernalization, sixteen seedlings of each line were transferred to plastic pots together with their Jiffypots™ and placed on balances on a gravimetric platform (Droughtspotter, Phenospex, Netherlands) which recorded pot weight at 30 min intervals, with precision irrigation. Plastic pots (240 mm high x 165 mm diameter) were filled with 3.5 kg dry weight of a soil mix (1: 1: 1, coco peat-based potting mix: clay loam paddock soil: sand), supplemented with a slow-release fertilizer and covered with a double layer of foam mesh to minimize evaporation. A metal frame was placed around each plant for support. The combinations of sampling dates, treatments and lines were randomized to pots in the glasshouse according to a triple-split-unit design with four biological replicates. Each replicate block was divided into two areas, each of which was, in turn, divided into two subareas. The two sampling dates (i.e. during treatment and at maturity) were randomized to the areas within replicates and the two treatments (i.e. drought or combined drought and heat) were randomized to the two subareas within each area. The lines were randomized to pairs of pots within an area so that NIL contrasting allele pairs occurred together. Pots were manually irrigated and adjusted to their target weight corresponding to 20% soil water content and -0.44 MPa (S1 Fig) during the first two weeks and then automatically irrigated whenever the pot weight dropped 0.5% below target weight. Temperatures in the glasshouse were set to 22/15 ^o^C day/night with a 16 hours day length period and supplemental LED lighting providing approximately 400 μmoles.m^2^.s^-1^ at the canopy.

#### Drought and heat treatment

Plants were grown under well-watered conditions (20% soil moisture) except for a 9-day drought period commencing three days after anthesis. All plants were scored every day, with treatment starting three days after the anthesis of each individual. Drought was imposed by lowering the target weight to 12% soil water content, corresponding to -0.72 MPa (S1 Fig). Half of the plants were additionally subjected to heat stress (combined drought and heat) by transferring them to a neighbouring glasshouse with 35/25 ^o^C day/night and similar light settings during the last three days of the drought period. During the heat treatment, plants were irrigated manually to the target weight four times a day. After nine days, plants were moved back to their spot on the gravimetric platform and kept under well-watered conditions until reaching physiological maturity. Transfer of pots and changes in treatments were carried out daily at 8:00 am to maintain a consistent treatment period. Water use, temperature and relative humidity were recorded at 30 min intervals in both gravimetric and neighbouring glasshouses throughout (S2 Fig).

### Plant phenotyping

Flowering time, defined as the first anther extrusion of the primary tiller, was scored daily for all plants. Four of the replicates of each line were used for physiological measurements during growth and harvested at maturity, while the other four were used for ribonucleic acid (RNA) sequence analysis (i.e. sampling during treatment). Hyperspectral reflectance measurements (350–2500 nm) were taken daily between 10:30 am and 12:00 pm in the centre of the flag leaf of the primary tiller from anthesis till three days after treatment (0–14 days after anthesis, DAA) using the ASD FieldSpec 3 portable spectroradiometer (Malvern Panalytical, United Kingdom). To transform the obtained wavelength into values of physiological traits, the data were uploaded to the wheat physiology predictor website [22–24]. Physiological traits obtained from hyperspectral measurements included leaf dry mass per area (representing leaf thickness), chlorophyll index, photosynthetic capacity (normalized to 25°C), electron transport capacity and mitochondrial respiration rate (normalized to leaf dry mass).

At physiological maturity, plant height of the primary tiller of the four replicates of each NIL was measured as the distance between the base of the stem and the top of the spike excluding awns. Spike length was measured from the base of the first spikelet to the tip of the last spikelet of the primary tiller. The number of spikes per plant was counted. Samples were dried at 37 ^o^C for 10 days and total above-ground biomass per plant was determined including stems, leaves and spikes of all tillers. Grain traits per primary tiller and for the remainder of the spikes were analysed separately. To differentiate between small (< 2.0 mm) and large grains (> 2.0 mm) a wheat grain sieve (2.0 mm, Graintec, Australia) was used. Grain weight and grain number were determined for grains > 2.0 mm. Single grain weight was measured as the average of grain weight divided by the number of grains. Screenings was defined as the difference in percentage of small grains (< 2.0 mm) compared to total grain weight.

### Statistical data analysis of phenotypic traits

Linear mixed models analyses of the phenotyping data were conducted using the R packages ASReml [25] and asremlPlus [26], with the data for the screenings trait being transformed so that they were normally distributed. The models included random terms for the subareas and pairs of pots that were part of the experimental design and allowed for unequal variances between pots for the combinations of treatments and families. Statistical tests were conducted to assess whether these models could be simplified. Also, for each trait, the differences between the combinations of the two NILs of interest here and the treatments were investigated by choosing a model to describe them based on the P-values calculated for both the NIL and treatment main effects and for their interaction. Predicted means (BLUEs) and least significant differences for a 5% level of significance [LSD (5%)] were obtained under the chosen model for the four NIL-treatment combinations. The average daily water consumption was estimated over three consecutive multiple-days periods: during the first six days of drought (3–8 DAA), during the last three days of drought or combined drought and heat stress (9–11 DAA) and during recovery (12–42 DAA). The daily water use index was calculated by dividing the above-ground biomass measured at maturity by the average daily water consumption during these periods. We used the term ‘water use index’ rather than ‘water use efficiency’ because, although we reduced the evaporation during the experiment to a minimum, it is not directly measured using the gravimetric platform. Physiological traits were assessed across the same three time periods as the water consumption and, in addition, for the pre-treatment period (0–2 DAA).

### Gene expression analysis by RNA sequencing

#### Sampling and RNA isolation

The first two spikelets of the primary tiller with extruding anthers of four independent plants per genotype were marked at anthesis. For RNA sequencing, two developing grains from the two spikelets were collected on the fifth day of drought treatment (i.e. eight days after anthesis (8 DAA), spikelet number one) and on the last day of either drought or combined drought and heat treatment (i.e. 11 days after anthesis (11 DAA), spikelet number two), snap frozen using liquid nitrogen and stored at -80 ^o^C. All samples were collected between 10:00 and 11:00 am. A total of four replicates per line, treatment and time point were collected. RNA was isolated using the Spectrum Plant Total RNA kit (Sigma-Aldrich‎, United States) including alpha-amylase (E-BLAAM 54.0 U/mg, Megazyme, United States) and DNAse (On-Column DNase I Digestion Set, Sigma-Aldrich‎, United States) treatments. Samples were further purified using the RNA Clean & Concentrator-5 kit (Zymo Research‎, United States) according to the manufacturer’s instructions. Samples were subsequently sent for quality check (Agilent Bioanalyzer 2100, Agilent Technologies, United States) and concentration measurement (Qubit 2.0 fluorometer, Thermo Fisher Scientific, United States) to the ACRF Cancer Genomics Facility (Adelaide, Australia). 15 samples with RNA integrity number (RIN) of at least 8.9 collected at 11 DAA (four replicates per treatment and line, except for one sample where RNA extraction failed) and eight samples collected 8 DAA were sent for sequencing and data analysis to NovoGene (Beijing, China). One additional sample collected 8 DAA (ID: B23, sample name: D8_AA4) was included because it differed in its phenotype from the other replicates (i.e. reduced plant height and biomass).

#### RNA sequencing

Following quality check of the RNAs, mRNA was enriched using oligo beads, followed by a random fragmentation and a single and then double stranded complementary deoxyribonucleic acid (cDNA) synthesis. Further steps including purification, terminal repair, poly-A-tailing, ligation of sequencing adapters, fragments selection and polymerase chain reaction (PCR) enrichment were performed to obtain the final cDNA libraries. Library concentration was first quantified using a Qubit 2.0 fluorometer and then diluted to 1 ng/μl before checking insert size on an Agilent Bioanalyzer 2100 and quantifying to greater accuracy by quantitative PCR. RNA sequences were obtained through the Illumina sequencing platform (Illumina, United States).

#### Data analysis

Raw data output from Illumina were transformed to sequence reads by base calling and recorded in a FASTQ file. Initial quality checks included an estimation of error rates for each base along reads and guanine-cytosine content distribution, followed by the removal of reads containing adapters or which were of low quality. After the quality checks, sequences were mapped against the IWGSC reference genome version 2 (https://wheat-urgi.versailles.inra.fr/Seq-Repository/Assemblies) using the hierarchical indexing for spliced alignment of transcripts (HISAT) software [27]. Total gene expression was estimated by counting the reads that mapped to genes or exons. Thereby, read count was not only proportional to the actual gene expression level, but also to the gene length and sequencing depth. In order to compare gene expression levels of different genes, the fragments per kilobase of transcript sequence per millions base pairs sequenced (FPKM) were calculated, taking into account the effects of both sequencing depth and gene length [28]. Gene expression levels were analysed using the HTSeq software [29]. Pearson correlations were calculated to reveal differences in gene expression between samples. Differences in gene expression between treatments, timepoints and alleles including all biological replicates were estimated using the software DESeq [30, 31]. The resulting P-values were adjusted using the Benjamini and Hochberg’s approach for controlling the false discovery rate. Genes with an adjusted P-value <0.05 were assigned as differentially expressed.

Genes with similar expression patterns were clustered together and represented in a heat plot. Gene ontology (http://www.geneontology.org/) enrichment analysis of differentially expressed genes was carried out using the GOseq software [32] based on the Wallenius non-central hyper-geometric distribution. To assign the differentially expressed genes to their putative biological function and pathway, a Kyoto Encyclopedia of Genes and Genomes (KEGG, https://www.genome.jp/kegg/kegg2.html) pathway enrichment analysis was conducted using *Oryza sativa japonica* as a reference genome. We used KOBAS software to test the statistical enrichment of differential expression genes in KEGG pathways. Genes which were identified as differentially expressed between NILs across treatments and timepoints were aligned to the International Wheat Genome Sequencing Consortium (IWGSC) Chinese Spring (CS) RefSeq v1.0 [20] using BLASTN with an e-value cutoff of 10^−40^ in order to find their putative locations in the wheat genome.

## Results

### Genotyping by sequencing of near-isogenic lines

Near-isogenic lines (NILs) were developed targeting a QTL interval of ~7 Mbp on the long arm of chromosome 6B. tGBS data of the five NIL pairs at BC1F6 (Fig 1, S2 Table) indicated 93.7% similarity between NILs and a ~17 Mbp interval, including the target region, segregating between NIL pairs on chromosome 6B (RefSeq v1.0). Further segregating regions were detected on chromosomes 1B and 7A. NILs carrying the non-exotic allele (Gladius) were 95.7% similar to each other and segregated on chromosomes 1B and 4A. NILs carrying the exotic allele from Taferstat were heterozygous for regions on chromosomes 1B, 3B and 7A with a genotypic similarity of 96.1%. As expected, NILs segregating for the target region were genotypically more different than replicates of the same NIL. Genetic differences between replicates (3.7–4.5%) could be due to residual heterozygosity of the plants which were selected and separately propagated from BC1F5.

### Plant growth conditions

Environmental conditions were fairly consistent during the vegetative stage and the crucial experimental period of July and August (-50 to 45 DAA) with maximum and minimum temperatures of 22.0–24.9 and 13.4–15.8 ^o^C during day and night, respectively, in the unheated treatment (S2 Fig). Maximum temperatures increased towards the end of the experiment up to 30 ^o^C, when plants were maturing (from 48 DAA onwards). Temperatures during the heat treatment reached 33.5–35.3 ^o^C maximum during the day and 23.5–24.5 ^o^C minimum at night. On average, pots reached the targeted drought level of 12% soil water content after 3.5 days of withholding irrigation.

### Phenotypic data

#### Yield-related traits

**Fig 2:**
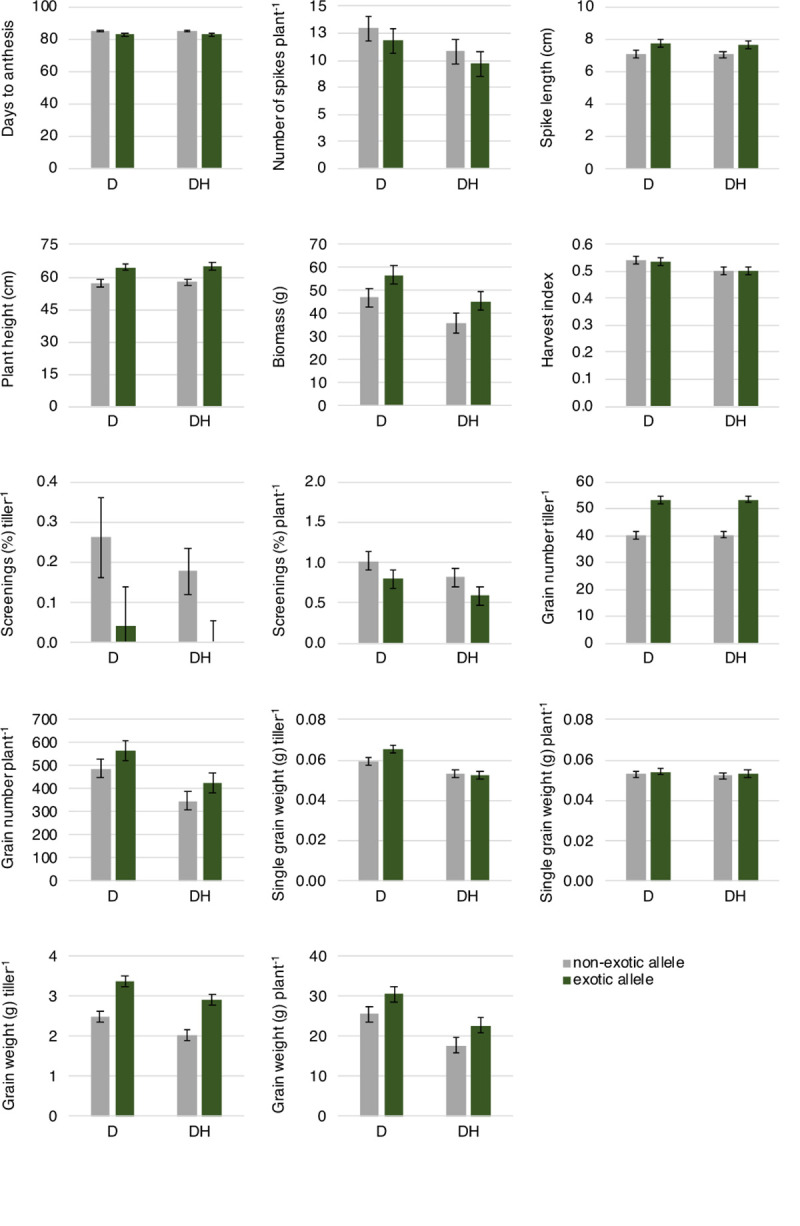
Yield-related traits of near-isogenic lines carrying the non-exotic (grey) or exotic (green) allele at the 6B QTL under drought and combined drought and heat. The error bars are ± half-LSD (5%), with error bars for different predicted means that overlap indicating the differences are not significant; those that do not overlap are significantly different. n = 4 BC1F6 plants per NIL. Tiller refers to the primary tiller of the plant. D, drought; DH, drought and heat stress.

Flowering time occurred over a time frame of 13 days with the first plant flowering 80 days after sowing. NILs carrying the exotic allele at the target region on chromosome 6B flowered, on average, two days earlier (p = 0.011) in comparison to NILs carrying the non-exotic allele (Fig 2). Similarly, plant height and biomass were promoted by the exotic allele under drought and combined drought and heat (p ≤ 0.022) with average increases of 7.5 cm in length and 0.6 g in weight. Biomass, harvest index and grain weight were reduced under combined drought and heat stress in comparison to drought in both NILs (p ≤ 0.05). Single grain weight was reduced in the primary tiller under combined drought and heat stress compared to drought, but similar under both stresses when measured per plant. In contrast, grain number was reduced under drought and heat stress per plant but not per primary tiller. Spike length, spike number, plant height and screenings were similar in both treatments.

Significant differences between alleles at the 6B QTL were found in six of the eleven yield component traits (Fig 2). Grain weight of the primary tiller and whole plant was increased by the allele derived from Taferstat in both treatments (p ≤ 0.011) and, in addition, screenings per plant were reduced in NILs carrying this allele (p ≤ 0.03) in both treatments. Grain number per primary tiller was increased in NILs carrying the exotic allele in both treatments, a potential result of the longer spikes in NILs carrying the exotic allele. Grain number per plant showed the same trend but differences were not significant. Similarly, single grain weight was significantly different under drought between both NILs per primary tiller, but not per plant.

#### Water use

**Fig 3:**
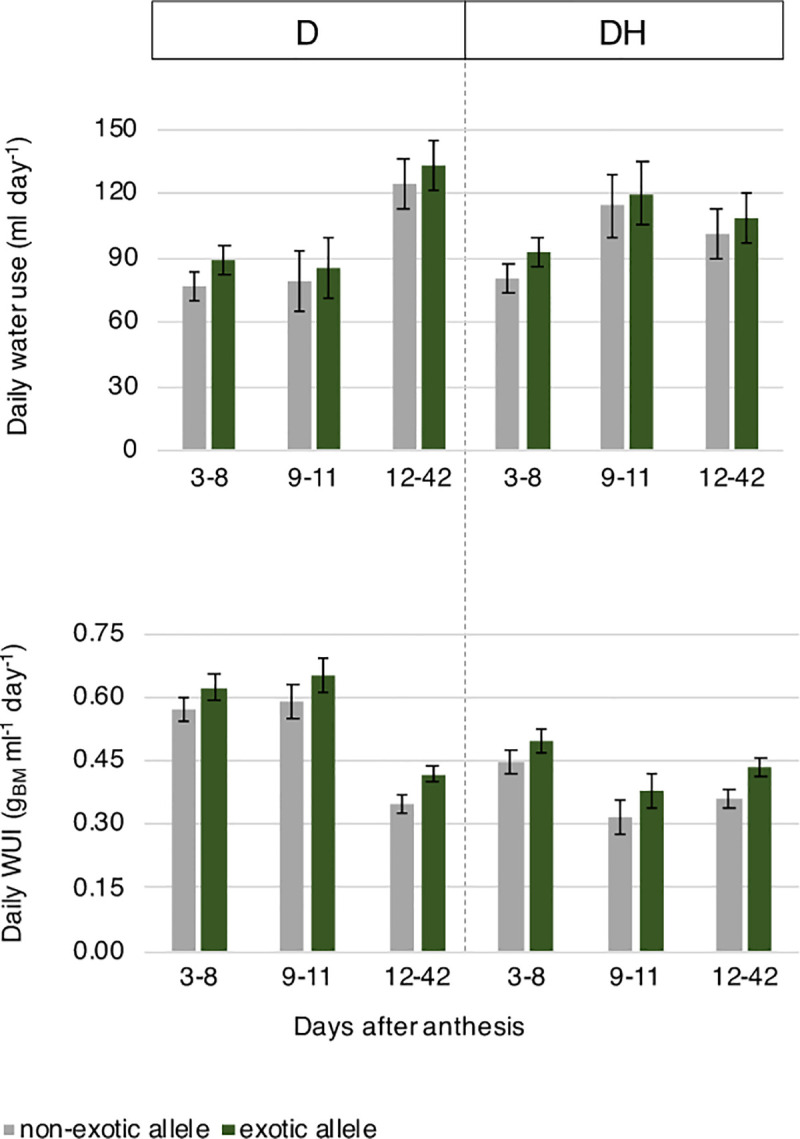
Predicted means of daily water use and daily Water Use Index (WUI) of near-isogenic lines under drought and combined drought and heat stress. Grey = the non-exotic allele, green = exotic allele. The error bars are ± half-LSD (5%), with error bars for different predicted means that overlap indicating the differences are not significant; those that do not overlap are significantly different. n = 4 BC1F6 plants per NIL. D, drought; DH, drought and heat stress; g_BM_, above-ground biomass at maturity (g).

The average daily water use did not differ significantly between the treatments during the drought treatment period and was 84.7 ml per day (Fig 3). For the drought treatment the average daily water use did not increase as the treatment prolonged (i.e., 9–11 DAA), being 82.1 ml per day, but it increased by 46.8 ml per day during the recovery period. On the other hand, the average daily water use increased markedly during the combined drought and heat treatment, when it was 117.1 ml per day; it then remained little changed during the recovery period at 104.64 ml per day. On average, plants under drought stress decreased their water use 52 DAA, i.e. 41 days after treatment, whereas plants under combined drought and heat stress reduced their water use 36 days after treatment. The daily water use was, however, similar in both NILs with NILs carrying the exotic allele tending to have a slighter higher water use throughout the experiment.

Daily water use index ranged between 0.57 and 0.62 g biomass ml^-1^ day^-1^ during the drought treatment (3–11 days, Fig 3) but was reduced in plants subject to combined drought and heat treatment and during recovery (0.32–0.50 and 0.35–0.43 g biomass ml^-1^ day^-1^, respectively). During recovery (12–42 days), daily water use index was increased in NILs carrying the exotic allele (p = 0.002) compared with NIL carrying the non-exotic allele. Similar to the water consumption, no significant differences between NILs prior to and during the treatment were observed, although NILs carrying the exotic allele tended to have a higher water use index.

#### Photosynthesis-related traits

**Fig 4:**
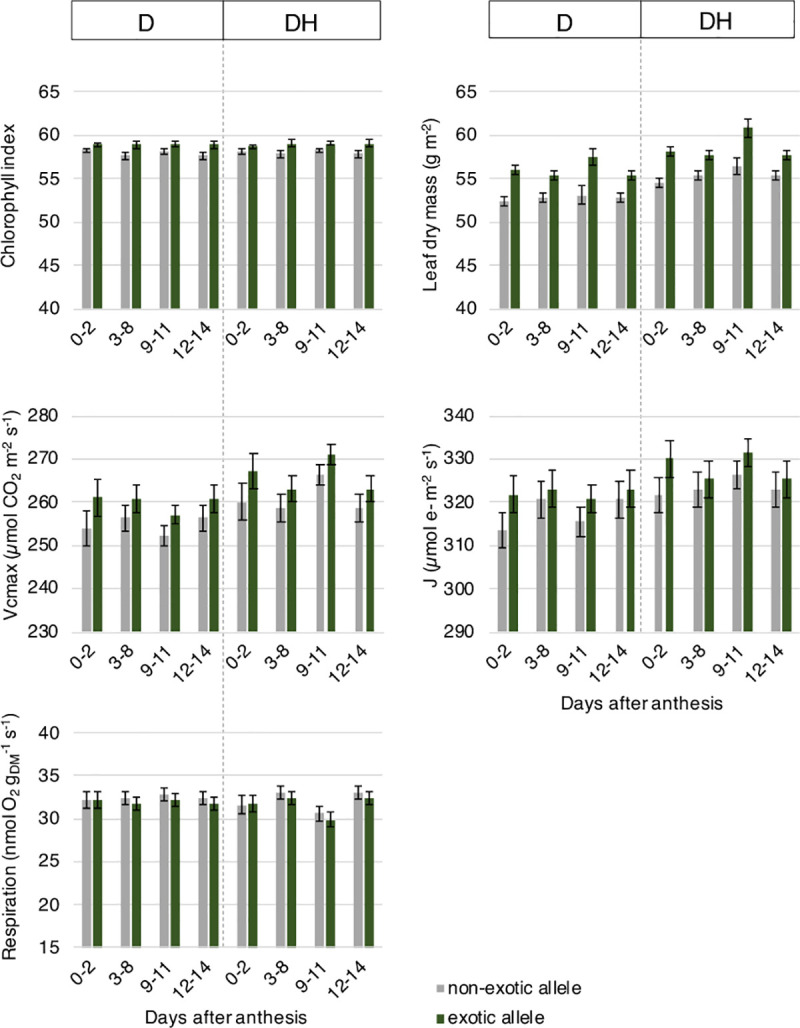
**Photosynthesis-related traits of near-isogenic lines carrying the non-exotic (grey) or exotic (green) allele at the target region on chromosome 6B under drought and combined drought and heat.** The error bars are ± half-LSD (5%), with error bars for different predicted means that overlap indicating the differences are not significant; those that do not overlap are significantly different. n = 4 BC1F6 plants per NIL. D, drought; DH, drought and heat stress; g_DM_, g dry mass; J, electron transport capacity; Vcmax, photosynthetic capacity.

Photosynthesis-related traits were relatively stable throughout the whole measurement period in both NILs and treatments, except for an increase (p ≤ 0.005) in leaf dry mass, photosynthetic capacity (Vcmax) and electron transport capacity (J) and a decline (p = 0.013) in the respiration rate under combined drought and heat compared to drought alone at 9–11 DAA (Fig 4). NILs carrying the exotic allele had increased leaf dry mass per area (i.e., thicker leaves) and chlorophyll index in comparison to NILs carrying the non-exotic allele at all times and in both treatments (p ≤ 0.05). Photosynthetic and electron transport capacity showed a similar trend but were only significantly higher in lines carrying the exotic allele at 9–11 DAA (p = 0.038) and 0–2 DAA (p = 0.045), respectively. Mitochondrial respiration rate was similar over the measurement period with no significant differences between NILs.

### Gene expression analysis of developing grains

RNA sequencing data were of good quality with 94.7–99.0% of clean data in each sample. The percentage of mappable reads for all samples was above 70% (S5 Table). Most reads could be mapped to exons (57.6–76.2%), followed by intergenic regions (22.5–41.5%). The smallest proportion was mapped to intron regions (0.8–2.0%). Some of the reads were mapped to more than one exon (17.5–26.3%) potentially due to repetitive DNA within a chromosome or a gene copy on one of the other two homeologous chromosomes. Read densities were similar for the positive and negative strands of each chromosome.

Overall, all samples showed similar gene expression levels with the majority of genes poorly expressed (FPKM <1 for ≥55.9% of the total number of genes) (S6 Table). Genes with a medium to high expression, i.e., FPKM between 1–3, 3–15, 15–60, accounted for 11.3–13.2%, 19.3–21.7% and 6.1–8.0% of the genes, respectively. Genes with a very high expression (FPKM > 60) accounted for 1.8–2.3% of the genes. Biological replicates were 90–99% similar in their gene expression (S7 Table), except for sample “D8_AA4”. The sample “D8_AA4” was included as additional sample because of its phenotypic difference (i.e., reduced plant height and biomass) in comparison to the other replicates. Pearson correlations (R^2^) between “D8_AA4” and the other replicates collected at 8 DAA carrying the non-exotic alleles ranged between 0.79 and 0.81, at the limit of the suggested threshold of 0.80 for reliable replicates. tGBS data did not reveal any difference in the genome of plants with reduced biomass and plant height in comparison to plants showing the common phenotype. The markers used for tGBS might not have covered the region associated with this phenotype, or an epigenetic modification might have caused this difference.

**Fig 5:**
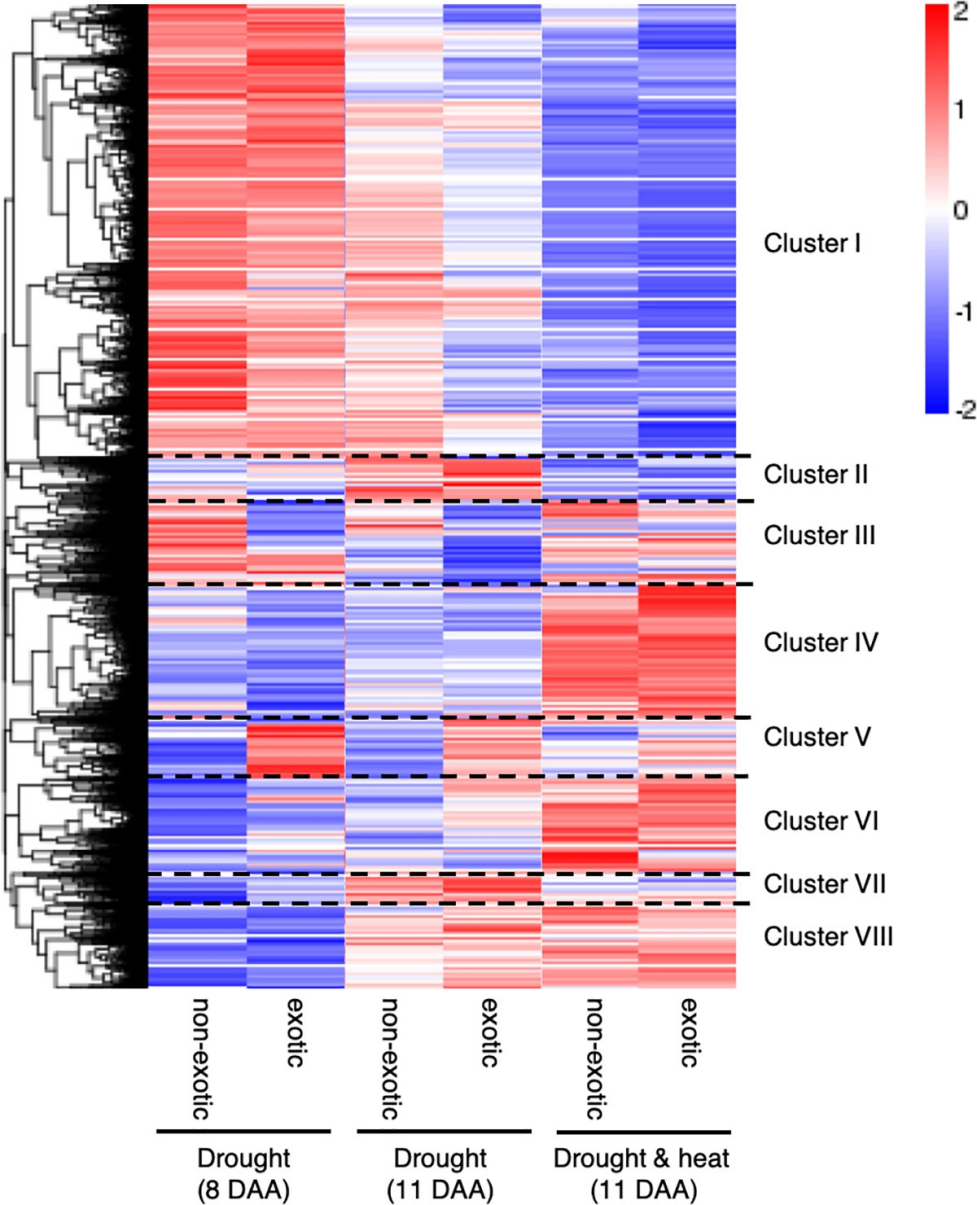
Hierarchical cluster analysis of differentially expressed genes in developing grains of near-isogenic lines under drought and combined heat (sourced from NovoGene). Samples were collected on the fifth day of treatment (i.e. 8 days after anthesis) when all plants were subjected to drought and on the last day of treatment (i.e. 11 days after anthesis) when plants were subjected to either drought or combined drought and heat stress. Red, upregulated genes (> 0); blue, downregulated genes (< 0). The colour range from red to blue represents the log_10_ (FPKM+1) value from large to small.

A total of 42,393 genes were similarly expressed in both NILs and treatments. Genes differentially expressed between the two treatments at 11 DAA (Fig 5 - clusters II, IV, VI and VII, S8 Table) or between timepoints (i.e., 8 DAA and 11 DAA) (Fig 5 - clusters I and VIII, S8 Table) accounted for 5,507 and 28,371 of the genes, respectively. Of these, 2,278 and 10,579 were differentially expressed between treatment or timepoint in both NILs. Genes differing between timepoints were principally involved in plant and grain development such as cell number regulation, DNA repair mechanisms and transport and hydrolysis of sugars (S9 and S10 Tables).

**Fig 6:**
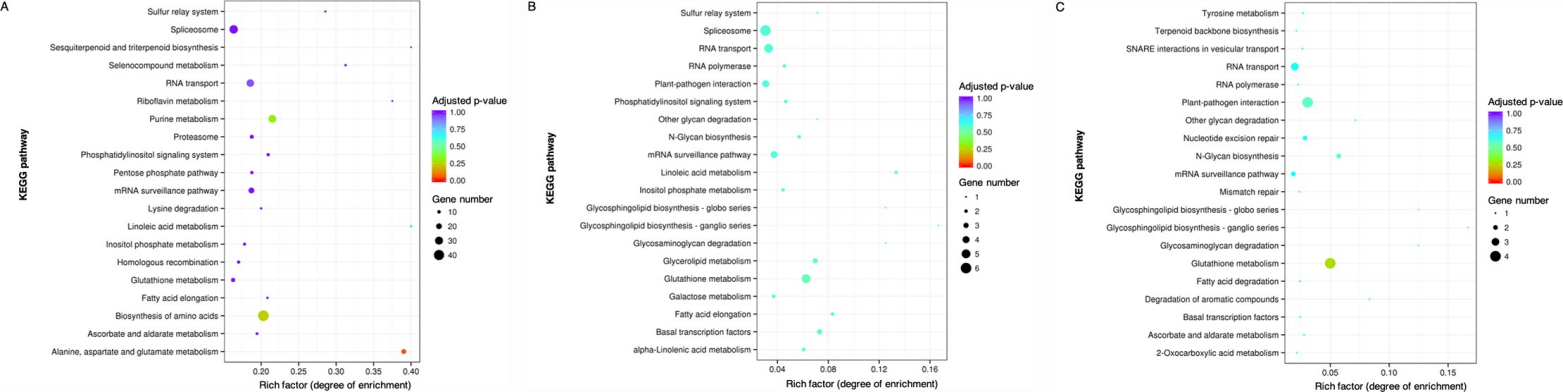
Kyoto Encyclopedia of Genes and Genomes (KEGG) enrichment analysis of differentially expressed genes in developing grains of near-isogenic line allele pairs under drought at 8 days after anthesis (A), under drought at 11 days after anthesis (B) and under combined drought and heat stress at 11 days after anthesis (C) (sourced from NovoGene). *, significantly enriched (adjusted p-value < 0.05).

NILs carrying the exotic allele differed by 3–11% in their number of expressed genes to NILs carrying the non-exotic allele (S7 Table) with a total of 2,082, 358 and 164 differentially expressed genes under drought at 8 DAA, under drought at 11 DAA and under drought and heat at 11 DAA, respectively. Differentially expressed genes at 8 DAA were mainly located on the long arms of chromosomes 1B (64 genes), 4B (1,037), 6B (37) and 7A (18) and on the short arm of chromosome 4B (763 genes) (S8 Table). Most of these genes encode proteins which are cellular components of the cytoplasm and the endomembrane system (S9 Table). Upregulated genes on chromosome 4B in NILs carrying the exotic allele were mostly involved in alanine, aspartate and glutamate metabolism (Fig 6A, S10 Table). Genes differently expressed between NILs at 11 DAA under drought were similar to those detected at 8 DAA and mostly, but not significantly, associated with cell components (Fig 6B). Differentially expressed genes under combined drought and heat at 11 DAA were dominantly located on the long arms of chromosomes 1B (51 genes), 6B (48 genes) and 7A (16 genes); only one of the genes was located on chromosome 4B (S8 Table). Most of these genes encode binding proteins which are involved in pathways such as glutathione metabolism, plant-pathogen interactions and RNA transport (not significant) (Fig 6C).

A total of 67 high confidence and 8 low confidence genes were differentially expressed between NILs carrying the opposite allele across treatments and timepoints, with 27 of the genes located on the long arms of chromosome 1B (physical positions 330,193,821–583,693,092 bp), 36 genes located on the long arm of chromosome 6B (703,288,889–720,510,790 bp), one gene located on the long arm of chromosome 6D (462,012,557–462,015,085 bp) as well as 3 and 8 genes located on the short (11,422,765–20,486,172 bp) and long arms (635,069,694–681,015,619 bp) of chromosome 7A, respectively (Fig 5 - cluster V, Table 1). Thirty five genes were upregulated and 40 downregulated in NILs carrying the exotic allele. Both, upregulated and downregulated genes, are involved in cell and gene regulation, protein binding, disease resistance, carbohydrate transport and metabolic pathways. In addition, two of the upregulated genes are associated with the arginine and proline metabolism (TraesCS6B02G456400) as well as with the development of anatomical structures (TraesCS1B02G269500), whereas one of the downregulated genes (TraesCS1B02G288900) is associated with the Golgi vesicle transport and three encode proteins located in the chloroplast.

**Table 1:** List of genes differently expressed between near-isogenic lines across treatments and timepoints.

Former gene accession (Ensembl Plants release 25)	Updated gene accession (Ensembl Plants release 47)	Chr	Position (bp)	Corresponding protein	Gene regulation	log2	adjusted p-value
Traes_1BL_58A450CB0	TraesCS1B02G356100LC.1	1B	330,055,731–330,057,688	-	down	inf	8.76E-62
Traes_1BL_927C3ED7B	TraesCS1B02G183200	1B	330,193,821–330,218,044	Myosin-8	down	7.62	4.91E-46
Traes_1BL_A54461172	TraesCS1B02G183700	1B	330,501,851–330,504,531	UPF0415 protein C7orf25 homolog	down	1.80	9.24E-08
Traes_1BL_63F0F0FAA	TraesCS1B02G186500	1B	333,595,520–333,597,852	LanC-like protein GCR2	down	4.17	1.15E-09
Traes_1BL_C80BB3B0D	TraesCS1B02G375500LC.1	1B	348,478,933–348,479,535	-	up	7.91	2.57E-25
Traes_1BL_6956EAF2D1	TraesCS1B02G207100	1B	374,328,133–374,330,340	60S ribosomal export protein NMD3	down	3.20	6.27E-30
Traes_1BL_337760BBE	TraesCS1B02G209600	1B	380,461,431–380,467,221	Nuclease related NERD	up	4.08	3.53E-17
Traes_1BL_42FAD5DBE	TraesCS1B02G216300	1B	392,107,116–392,111,653	MLO-like protein 9	up	1.41	1.29E-10
Traes_1BL_1AA872E89	TraesCS1B02G250600	1B	442,622,871–442,626,924	Probable nitronate monooxygenase	down	2.11	4.08E-05
Traes_1BL_9A1A32022	TraesCS1B02G253700	1B	447,323,125–447,327,142	Protein N-terminal asparagine/ glutamine amidohydrolase	up	5.46	7.98E-16
Traes_1BL_D261AE149	TraesCS1B02G466000LC	1B	456,154,491–456,159,954	26S protease regulatory subunit 10B homolog A	up	8.06	3.17E-122
Traes_1BL_2DF3E745A	TraesCS1B02G258800	1B	456,252,451–456,255,472	-	up	1.14	1.02E-03
Traes_1BL_942C3A057	TraesCS1B02G262200	1B	460,578,389–460,581,993	Bax inhibitorI1-like protein	up	2.35	2.04E-27
Traes_1BL_664BABA7A	TraesCS1B02G262600	1B	461,298,262–461,312,532	Probable manganese-transporting ATPase PDR2	up	0.94	1.00E-04
Traes_1BL_5C0E7A70E	TraesCS1B02G269500	1B	473,864,248–473,868,617	Isopentenyl-diphosphate Delta-isomerase	up	1.22	1.03E-03
Traes_1BL_A762B63A2	TraesCS1B02G272900	1B	478,721,054–478,725,344	Protein root UVB sensitive 6	up	7.57	6.59E-152
Traes_1BL_9AE83440A	TraesCS1B02G279200	1B	487,489,192–487,496,862	CRAL-TRIO lipid binding	up	1.92	9.13E-08
Traes_1BL_BE03D01EF	TraesCS1B02G492100LC	1B	488,037,013–488,038,655	Uncharacterized protein	up	2.81	9.09E-33
Traes_1BL_72FF5A24C	TraesCS1B02G288900	1B	503,622,726–503,624,114	Yos-1 like	up	4.88	1.89E-69
Traes_1BL_4C3C75D3A	TraesCS1B02G289100	1B	503,871,963–503,881,842	Ankyrin-1	down	5.08	1.67E-36
Traes_1BL_7BB61786E	TraesCS1B02G291200	1B	508,553,015–508,555,855	Aldo-keto reductase family 4 member C9	up	1.65	1.91E-09
Traes_1BL_3CB12266D	TraesCS1B02G291500	1B	508,707,538–508,709,360	Aldo-keto reductase family 4 member C10	down	3.21	2.93E-24
Traes_1BL_28B2386DF	TraesCS1B02G292400	1B	509,891,166–509,892,675	Peptidyl-prolyl cis-trans isomerase	down	3.13	7.10E-35
Traes_1BL_2B7961A65	TraesCS1B02G292800	1B	510,598,733–510,602,454	Copper transport protein CCH	up	2.25	2.07E-17
Traes_1BL_9C2B2A24A	TraesCS1B02G298500	1B	520,083,939–520,091,297	Serine/threonine-protein kinase ATR	down	3.08	1.72E-22
Traes_1BL_8FABA743F	TraesCS1B02G559300LC	1B	549,463,918–549,465,895	-	down	4.06	3.31E-19
Traes_1BL_C5C66A768	TraesCS1B02G353500	1B	583,689,036–583,693,092	Probable peptidyl-tRNA hydrolase 2	up	4.42	4.71E-28
Traes_6BL_81481E353	TraesCS6B02G435000	6B	703,288,889–703,290,737	Mitochondrial import protein TIM15	down	7.10	5.28E-14
Traes_6BL_882745936	TraesCS6B02G779400LC	6B	703,941,450–703,945,001		down	1.23	1.94E-06
Traes_6BL_93357D848	TraesCS6B02G436400	6B	704,038,332–704,042,869	Serine/threonine-protein phosphatase PP1	down	0.85	7.06E-03
Traes_6BL_5AF540208	TraesCS6B02G436600	6B	704,153,686–704,156,343	F-box LRR protein	down	2.55	6.18E-05
Traes_6BL_F914C4C3A	TraesCS6B02G437000	6B	704,187,356–704,189,481	Putative ribonuclease H protein At1g65750	down	5.14	9.00E-34
Traes_6BL_A0879077D	TraesCS6B02G783000LC.1	6B	704,949,053–704,949,315	T-complex protein 1 subunit zeta 1	down	1.01	1.96E-05
Traes_6BL_0CB2299E9	TraesCS6B02G437200	6B	712,227,427–712,229,904	-	down	7.80	6.42E-47
Traes_6BL_4704FF2B4	TraesCS6B02G440200	6B	705,377,867–705,385,342	Putative aconitate hydratase, cytoplasmic	down	2.77	5.41E-29
Traes_6BL_FBA80D37C	TraesCS6B02G440500	6B	705,497,114–705,500,519	Probable mediator of RNA polymerase II transcription subunit 36b	down	1.65	2.84E-12
Traes_6BL_B55E3EF6A	TraesCS6B02G441200	6B	705,613,631–705,620,958	Putative disease resistance protein RGA3	down	inf	3.98E-23
Traes_6BL_CD8C2012C1	TraesCS6B02G441400	6B	705,634,389–705,636,966	Probably inactive leucine-rich repeat receptor-like protein kinase IMK2	down	inf	5.66E-09
Traes_6BL_616BBA730	TraesCS6B02G442200	6B	705,885,467–705,886–688	Disease resistance protein RGA2	down	inf	3.43E-26
Traes_6BL_9E98E2626	TraesCS6B02G443700	6B	706,625,650–706,628,754	ATG8-interacting protein 1	down	4.25	9.48E-13
Traes_6BL_55445DC2D	TraesCS6B02G451500	6B	710,149,424–710,152,200	F-box protein	up	4.65	5.09E-38
Traes_6BL_B84BC12EC	TraesCS6B02G452100	6B	710,795,550–710,799,389	-	up	8.84	4.72E-190
Traes_6BL_4C6B99385	TraesCS6B02G452500	6B	711,345,338–711,348,401	Telomere length and silencing protein	up	3.72	2.31E-19
Traes_6BL_55A1538E1	TraesCS6B02G452700	6B	711,372,963–711,375,917	Telomere length and silencing protein 1	up	1.89	3.52E-08
Traes_6BL_E8F3B2953	TraesCS6B02G453300	6B	711,709,311–711,712,023	F-box like protein	up	3.95	4.68E-11
Traes_6BL_7DB204621	TraesCS6B02G454800	6B	712,227,427–712,229,904	L-type lectin-domain containing receptor kinase IV.4	up	3.72	1.67E-02
Traes_6BL_CED3DB508	TraesCS6B02G456000	6B	712,345,436–712,351,600	ABC transporter F family member 3	up	1.85	6.41E-17
Traes_6BL_C48F853F8	TraesCS6B02G456400	6B	712,658,222–712,662,336	Probable prolyl 4-hydroxylase 3	up	2.67	9.19E-19
Traes_6BL_FAD2FD89C	TraesCS6B02G456500	6B	712,665,811–712,674,449	Sucrose transport protein SUT4	up	1.16	1.35E-04
Traes_6BL_E204B7C91	TraesCS6B02G457400	6B	713,114,509–713,117–699	NB-ARC-LRR	up	1.52	5.97E-03
Traes_6BL_FF26CBFBF	TraesCS6B02G460100	6B	713,971,067–713,976,790	Synaptotagmin-like 1 homologous	up	5.84	1.70E-41
Traes_6BL_EE1166E22	TraesCS6B02G465800	6B	716,361,322–716,368,720	Disease resistance protein RPP13	down	inf	2.12E-19
Traes_6BL_7C8EDCF5A	TraesCS6B02G466700	6B	716,622,427–716,628,907	Protein argonaute 1C	down	6.52	6.27E-30
Traes_6BL_2D48C932A	TraesCS6B02G468200	6B	717,861,512–717,874,664	Callose synthase 3	down	1.85	7.33E-18
Traes_6BL_20CA191B4	TraesCS6B02G468300	6B	717,891,358–717,892,813	Outer envelope pore protein 21, chloroplastic	down	1.75	7.57E-08
Traes_6BL_C8ED6D1AF	TraesCS6B02G468600	6B	717,9151,50–717,919,160	Callose synthase 3	down	5.70	8.93E-17
Traes_6BL_1787AAD1C	TraesCS6B02G468900	6B	717,961,216–717,964,216	Metal tolerance protein C2	down	3.69	3.27E-12
Traes_6BL_DF9519C97	TraesCS6B02G470000	6B	718,382,723–718,387,419	Alpha-1,3-mannosyl-glycoprotein 2-beta-N-acetylglucosaminyltransferase	down	0.96	5.48E-04
Traes_6BL_077C91EAE	TraesCS6B02G470700	6B	718,765,354–718,775,528	Protein DETOXIFICATION 45, chloroplastic	down	1.77	1.28E-09
Traes_6BL_5E211AB35	TraesCS6B02G470800	6B	718,919,952–718,923,804	Unknown function	down	0.77	9.79E-03
Traes_6BL_CBDCDEFC5	TraesCS6B02G471700	6B	719,709,634–719,725,528	Protein Furry/ Tao3/ Mor2	down	2.71	5.02E-17
Traes_6BL_71ABF50AC	TraesCS6B02G472700	6B	720,418,530–720,423,497	DEAD-box ATP-dependent RNA helicase 2	down	2.15	1.25E-14
Traes_6BL_ECEBFE4C1	TraesCS6B02G473000	6B	720,505,144–720,510,790	Organellar oligopeptidase A, chloroplastic/mitochondrial	down	2.38	4.71E-28
Traes_6DL_F06F9895B	TraesCS6D02G381700	6D	462,012,557–462,015,085	F-box protein	up	8.14	1.26E-112
Traes_7AS_1DF12C790	TraesCS7A02G031300LC	7A	11,423,423–11,424,188	OTU domain-containing protein At3g57810	down	8.36	2.72E-29
Traes_7AS_D4796D360	TraesCS7A02G040000	7A	18,054,572–18,055,221	Disease resistance RPP8-like protein 3	up	9.64	6.78E-05
Traes_7AS_73D26354E	TraesCS7A02G044300	7A	20,482,829–20,486,172	Disease resistance protein RPM1	up	10.36	2.12E-06
Traes_7AL_2A57956BC	TreasCS7A02G440700	7A	635,069,180–635,069,850	Clathrin heavy chain 1-like	up	7.02	4.57E-06
Traes_7AL_E58674B35	TraesCS7A02G442500	7A	636,889,761–636,906,158	Glutathione synthetase, chloroplastic	up	7.74	3.56E-31
Traes_7AL_060F5996C	TraesCS7A02G448300	7A	642,731,873–642,735,937	26S protease regulatory subunit S10B homolog	up	8.22	2.15E-105
Traes_7AL_6E220ADA0	TraesCS7A02G457200	7A	652,703,802–652,712,442	Mechanosensitive ion channel protein 2, chloroplastic	down	4.31	3.57E-07
Traes_7AL_879EF12ED	TraesCS7A02G479800	7A	672,024,985–672,029,520	Eukaryotic translation initiation factor 5B	up	4.02	2.60E-40
Traes_7AL_1A15283C9	TraesCS7A02G484800	7A	675,587,045–675,590,716	G-type lectin S-receptor-like serine/threonine-protein kinase B120	down	9.11	3.65E-12
Traes_7AL_47158BA7C	TraesCS7A02G491300	7A	680,341,311–680,345,280	Ubiquitin-conjugating enzyme E2	up	1.81	1.26E-07
Traes_7AL_B8CD6C57F	TraesCS7A02G492400	7A	681,011,336–681,015,619	Probable auxin efflux carrier component 2	down	3.27	1.69E-08

Positions in base pairs (bp) are based on the RefSeq v2.0 (http://plants.ensembl.org/Triticum_aestivum/Info/Index). Chr, chromosome; down, downregulated genes in NILs carrying the exotic allele compared to the NIL with the non-exotic allele; inf, gene which was exclusively expressed in either NIL carrying the non-exotic or exotic allele; log_2_, fold change in gene expression; up, upregulated genes in NILs carrying the exotic allele. P-value corresponds to adjusted p-value.

## Discussion

The identification of QTL and the underlying genes associated with grain yield and yield stability following abiotic stress can be valuable for the development of new, high yielding varieties. Identified QTL and their associated molecular markers can be used for marker-assisted selection, a method which enables the selection of genotypes in large populations with reduced need for costly and time-consuming phenotyping in the field [33]. Knowing the genes and their function, on the other hand, can provide information on key mechanisms associated with stress tolerance and can be used for the direct modification of current cultivars by methods such as CRISPR [34]. Of similar importance is the physiological dissection of these QTL into their components, which can then serve as target traits for breeding in dry and hot climates. Using NILs, we studied a target QTL on the long arm of chromosome 6B and its effect on yield components, water use and photosynthesis-related traits. The QTL was previously identified in three independent studies [17–19], contributing to grain weight, single grain weight, harvest index and leaf chlorophyll content, but with opposite allelic effects under semi-controlled compared with field conditions.

NILs carrying the allele from the exotic parent at chromosome 6B had an increase in grain weight, grain number and single grain weight as well as a decrease in screenings. Results were therefore consistent with results observed in semi-controlled conditions [17]. Differences in grain number, single grain weight and screenings were significant per primary tiller but not per plant. Anthesis was recorded only for the primary tiller. Other tillers may have differed in their development compared to the primary tiller, varying the timepoint and impact of the stress, buffering thus the differences between NILs. Differences in the development of tillers could also explain the observed variation in yield components within the same plant. Single grain weight was more severely affected by the combined drought and heat stress at the primary tiller compared to drought alone, whereas grain number was more affected in the whole plant. Reductions in single grain weight are commonly associated with post-anthesis stress, whereas a decline in grain number is often observed following pre-anthesis stress [19, 35]. In our experiment, the stress treatments began three days after anthesis of the primary tiller, a post-anthesis stress. Final grain weights both per tiller and per plant were increased in plants carrying the exotic allele in both treatments. This indicated that the beneficial effect of the QTL was not dependent solely on post-anthesis growth and the development of tillers, but more likely related to either whole life cycle plant physiology or a change in grain filling or senescence, post-treatments.

Photosynthesis-related traits, including leaf dry mass, chlorophyll index, photosynthetic and electron transport capacity as well as mitochondrial respiration rate were fairly stable throughout the whole measurement period in both NILs, except for a peak during the combined drought and heat treatment. To avoid photoinhibition and allow acclimation, plants can optimise light absorption through leaf and chlorophyll movement and anthocyanin accumulation, as well as the energy balance of their photosystem, through modifications of CO_2_ fixation, photo- and mitochondrial respiration and cyclic electron flow [36, 37]. In our case, photosynthetic and electron transport capacity were reversibly modified in response to the occurrence and absence of the heat stress. This suggests that all NILs were able to acclimate to higher temperatures.

NILs carrying the exotic allele maintained their photosynthetic and electron transport capacity above NILs carrying the non-exotic allele with significant differences observed at 0–2 and 9–11 DAA. NILs carrying the exotic allele also had an increased leaf dry mass (i.e. thicker leaves) compared to NILs carrying the non-exotic allele before, during and after treatments, and increased chlorophyll index. A higher photosynthetic capacity would suggest a higher contribution to grain yield [38, 39], which was the case in our study.

Water use and photosynthesis-related traits have previously been suggested to be important traits to increase wheat yield potential under drought and heat stress [9, 13]. Particularly, an increased water use efficiency has often been hypothesized in literature to be associated with a higher stress tolerance [40–43], while others argue that the effective use of water (i.e. maximal soil moisture capture for transpiration) and not water use efficiency is important for crop improvement [12, 44].

In our case, water use was similar, although tendentially higher in NILs carrying the exotic allele, throughout the experiment. Both NILs adapted their water use equally to the changing water availabilities. Water use was increased in both NILs during the combined drought and heat treatment compared to drought alone, potentially due to an increase in transpiration for cooling. Pots were constantly re-irrigated to a certain target weight throughout the experiment instead of withholding irrigation completely, enabling a constant delivery of water. A higher water use and transpiration rate, therefore, had no negative effect in this experiment but might be a problem in a situation in which water availability was more restricted. Water use was also increased during the recovery phase following both treatments, likely due to the higher water availability, and resulting in an overall lower water use index during this phase. Although there were no differences between NILs for water use, NILs carrying the exotic allele had an overall higher water use index with a significant increase during recovery. Increased water use index in these NILs was due to increased biomass and this post-treatment difference suggested an accelerated stress-induced senescence in Gladius allele NILs compared with the exotic. The QTL at this locus was first identified for grain-filling heat stress tolerance for the ‘stay-green’ trait, i.e., delayed chlorophyll loss of the flag leaf [19]. Our results are consistent with these and underscore the importance of this trait. We additionally found increased leaf dry mass and leaf chlorophyll index throughout, including pre-stress, and in drought as well as combined drought and heat in the exotic NIL. The identification of the underlying physiological traits contributed by the beneficial alleles is particularly relevant to refine trait phenotyping priorities for growing environments where drought and heat stress occur simultaneously.

Segregation regions between NILs were observed not only at our target region on chromosomes 6B, but also on 1B and 7A. The region on chromosome 1B co-located on the Ref Seq v1.0 [20] with a previously identified QTL for anther extrusion in wheat [45]. The segregation region on chromosome 7A co-located with QTL identified for thousand kernel weight and spikelet number per spike [46–48]. One of our differentially expressed genes (TraesCS7A02G479800, Table 1), encoding a putative Eukaryotic translation initiation factor 5B, was located within the exact same region on the Ref Seq v1.0 as the previously identified QTL and was upregulated in NILs carrying the exotic allele in all treatments and timepoints. A second gene (TraesCS7A02G484800), encoding a G-type lectin S-receptor-like serine/threonine-protein kinase B120 was 1.3 Mb distant from the QTL and downregulated in NILs carrying the exotic allele. The increase in grain number and single grain weight in NILs carrying the exotic allele might have therefore been the result of the interaction of both QTL - 6B and 7A - or of one alone.

Most genes differentially expressed between NILs were observed at an early drought stage (i.e. 8 DAA), whereas this number decreased with an increase of stress duration (i.e. at 11 DAA) and intensity as shown under combined drought and heat treatment. Genes differentially expressed between NILs carrying the opposite allele were located on chromosomes 1B, 4B, 6B, 6D and 7A, i.e. mostly in regions of genotypic differences observed between NILs (Fig 1). Genotyping by sequencing data, however, did not suggest regions of segregation on chromosomes 4B and 6D. Genotypic differences might have had a trans-regulatory effect. Several of the differentially expressed genes between NILs, in particular those under drought and heat, were associated with regulation of gene expression and RNA processing supporting the hypothesis of a trans-acting control.

The majority of differentially expressed genes under drought were located on chromosome 4B with upregulated genes in NILs carrying the exotic allele being involved in alanine, aspartate and glutamate metabolism. All three amino acids have been observed to increase in developing grains of drought-resistant wheat plants when subjected to drought [49] and form part of the photorespiratory cycle [50, 51]. Differentially expressed genes under combined drought and heat stress were dominantly located on chromosomes 1B, 6B and 7A and were associated with the metabolism of glutathione, a component of the antioxidative system in plants, which is synthetised from glycine, a by-product of photorespiration [51]. Photorespiration, the fixation of O_2_ by ribulose-1,5- bisphosphate, is usually considered a negative trait in crop yield, degrading sugars which have been produced in energy-consuming reactions during photosynthesis, releasing CO_2_ and producing reactive oxygen species which can harm the cell. The ascorbate/ glutathione cycle is an important component in the regeneration of antioxidant scavengers and glutathione reductase and peroxidase have been previously observed to be specifically induced by drought and heat stress in tobacco [52].

A total of 36 differentially expressed genes across treatments and timepoints could be mapped to our target region on the long arm of chromosome 6B (Table 1). Several of these genes were associated with a wide range of functions such as carbohydrate transport, gene regulation, protein binding, disease resistance and various enzymatic activities, whereas others were of unknown function. To our knowledge, none of these genes has been previously characterized under drought or heat stress in wheat, except for one (TraesCS6B02G456400) whose predicted protein functions in hydroxylation of the well-studied amino acid proline. Proline has been shown to accumulate in plants in response to drought, heat and combined drought and heat stress [53, 54]. Its accumulation has been associated with tolerance mechanisms such as reactive-oxygen species scavenging, osmotic adjustment, signalling and the stabilisation of proteins [55, 56] as well as with an increase in grain yields [54, 57].

Seed weight was increased in NILs carrying the exotic allele by 24–32% under drought and by 6–23% under combined drought and heat stress. An increase of only 6% could mean, in theory, a contribution of 1 million tonnes per annum in Australia alone. Marker-assisted selection and introduction of such favourable alleles to breed higher yielding cultivars for regions affected by drought and heat stress is a promising approach. First steps have been achieved by introgressing the favourable allele into an Australian background in a nested-association mapping population [17]. However, with the selection for a single allele, such significant yield increases are less likely, as traits such as yield are under multigenic control and allelic effects are highly dependent on the environment. Nonetheless, this work is significant progress towards the identification of genes for drought and heat tolerance which can enable the discovery of new regulatory pathways in wheat and, possibly, the development of novel alleles via genome editing technologies.

We could not draw a conclusion about the opposite allele effects observed in the field by Garcia et al. [18] based on two trials in South Australia. However, performances in field trials are influenced by season to season environmental variations, additional and interacting biotic and abiotic stresses and, therefore, a number of field trials are required to confirm results. The higher harvest index promoted by the Australian allele in the field might have resulted from one of these factors rather than from actual drought or heat stress. On the other hand, a positive effect of the non-Australian allele was always found in pot experiments. Pot systems often differ in their water relations, the structure and temperature of the soil as well as the available root space and competition between plants compared to field conditions, all of which strongly influence root architecture and physiology as well as the interactions in the rhizosphere [58]. These factors could play a role in whether the exotic allele has a positive or negative effect on yield.

## Conclusions

Allelic effects of *QYld*.*aww-6B*.*1* on grain weight, single grain weight, grain number and screenings under drought and combined drought and heat stress were consistent with results from the genome-wide association study [17]. An increase in yield was also associated with thicker leaves, higher leaf chlorophyll index, a higher photosynthetic capacity and a higher water use index. Using gene expression analysis, we could narrow down our target region on chromosome 6B to 36 potential candidate genes with a further 39 genes of interest differentially expressed across treatments and timepoints in the NIL. Genes from four other regions within the genome (i.e., on chromosomes 1B, 4B, 6D and 7A) were differentially expressed between both NILs. Two of the four regions could be mapped to previously detected QTL, of which one has been identified for thousand kernel weight. Candidate differentially expressed genes were usually associated with genetic segregation, illustrating the value of the nested-association mapping population for the rapid incorporation and validation of the beneficial exotic alleles. The majority of these genes have not previously been associated with drought or heat stress tolerance in wheat and might serve as candidate genes for crop improvement in dry and hot climates. Further analysis regarding their involvement in the observed changes in physiology and yield components is required.

## Supporting information

S1 Fig Soil water potential–soil water content curve of the drought soil mix. Eight pots of the same size and filled with the same substrate mix as used in the experiment were first watered and then drained until reaching ~5% soil water content. The water content and water potential of the soil were measured daily by coring a of sample of 15 mm diameter and 200 mm length. The water content in soil was determined by calculating the weight difference between fresh soil sample and the oven-dried soil sample (at 65 ^o^C for 72 hours), divided by the oven-dried sample. The water potential of the fresh soil sample was measured using a water potential meter (WP4C, Meter Group, United States) in continuous mode until the value maintained stable. Dots, raw values of the eight pots. Blue line, logarithmic trendline.
(DOCX)

S2 Fig Daily temperature and relative humidity records in the DroughtSpotter glasshouse and the glasshouse used for the combined drought and heat treatment over the course of the experiment. The target temperatures were 22/15 ^o^C day/night and 35/25 ^o^C day/night in the DroughtSpotter and heated glasshouse, respectively. Anthesis date (i.e., 0 days after anthesis) represents the mean anthesis date. Relative humidity was not recorded from 18 to 22 days after anthesis and 33 to 41 days after anthesis due to a system failure.
(DOCX)

S1 Table KASP marker assisted selection for the development of near-isogenic lines. Grey indicates the allele derived from the non-exotic parent (Gladius) in the target regions of chromosome 6B. Green indicates the allele derived from the exotic parent (Taferstat).
(XLSX)

S2 Table Targeted genotyping by sequencing data of near-isogenic lines for the target QTL on chromosome 6B. The target region (T) and segregating regions (S) between NILs of the same pair as well as between replicates are marked in yellow. Exotic, allele from the Algerian parent (Taferstat); non-exotic, allele from the Australian parent (Gladius).
(XLSX)

S3 Table Quality and concentration of extracted RNA samples of near-isogenic lines. DAA, days after anthesis; QTL, quantitative trait locus. The phenotype of plants with ID E1 and B23 was slightly different (i.e., reduced plant height and biomass) from the rest of the replicates.
(XLSX)

S4 Table Quality assessment of the RNA sequencing data. Raw reads refer to the total number of reads, clean reads to the number of filtered reads. GC content, guanine-cytosine content; Q20, percentage of bases whose correct base recognition rates are greater than 99% in total bases. Q30, percentage of bases whose correct base recognition rates are greater than 99.9% in total bases.
(XLSX)

S5 Table Mapping of RNA sequences to the wheat genome. Total reads, total number of filtered reads (clean data); total mapped, total number of reads that could be mapped to the reference genome CS RefSeq v1; uniquely mapped, number of reads that were uniquely mapped to the reference genome; multiple mapped, number of reads that were mapped to multiple sites in the reference genome; reads map to '+', number of reads that map to the positive strand; reads map to '-', number of reads that map to the negative strand; exons, percentage of reads mapped to exons; introns, percentage of reads mapped to introns; intergenic, percentage of reads mapped to intergenic regions; non-splice reads, reads that were mapped entirely to a single exon; splice reads, reads that were mapped to two exons.
(XLSX)

S6 Table Total number of expressed genes in developing grains of Gladius, Taferstat and two NILs under either drought or combined drought and heat treatment. FPKM, fragments per kilobase of transcript sequence per million mapped reads. The higher the FPKM, the higher the gene expression.
(XLSX)

S7 Table Pearson correlation (R^2^) of number of expressed genes between samples. D8_AA, samples under drought collected 8 days after anthesis of NILs carrying the non-exotic allele AA (n = 5); D8_BB, samples under drought collected 8 days after anthesis of NILs carrying the exotic allele BB (n = 4); D11_AA, samples under drought collected 11 days after anthesis of NILs carrying the non-exotic allele (n = 3); D11_BB, samples under drought collected 11 days after anthesis of NILs carrying the exotic allele (n = 4); DH11_AA, samples under combined drought and heat collected 11 days after anthesis of NILs carrying the non-exotic allele (n = 4); D11_BB, samples under combined drought and heat treatment collected 11 days after anthesis of NILs carrying the exotic allele (n = 4).
(XLSX)

S8 Table Differently expressed genes comparing alleles, timepoints and treatments. -, no data available; D8_AA, samples under drought collected 8 days after anthesis of NILs carrying the non-exotic allele; D8_BB, samples under drought collected 8 days after anthesis of NILs carrying the exotic allele; D11_AA, samples under drought collected 11 days after anthesis of NILs carrying the non-exotic allele; D11_BB, samples under drought collected 11 days after anthesis of NILs carrying the exotic allele; DH11_AA, samples under combined drought and heat collected 11 days after anthesis of NILs carrying the non-exotic allele; D11_BB, samples under combined drought and heat treatment collected 11 days after anthesis of NILs carrying the exotic allele; log2, magnitude (fold-change) of gene expression.
(XLSX)

S9 Table Gene ontology enrichment analysis of differentially expressed genes. Statistical test method: hypergeometric test / Fisher's exact test. -, no significant enrichment; FDR, false discovery rate; D8_AA, samples under drought collected 8 days after anthesis of NILs carrying the non-exotic allele; D8_BB, samples under drought collected 8 days after anthesis of NILs carrying the exotic allele; D11_AA, samples under drought collected 11 days after anthesis of NILs carrying the non-exotic allele; D11_BB, samples under drought collected 11 days after anthesis of NILs carrying the exotic allele; DH11_AA, samples under combined drought and heat collected 11 days after anthesis of NILs carrying the non-exotic allele; D11_BB, samples under combined drought and heat treatment collected 11 days after anthesis of NILs carrying the exotic allele.
(XLSX)

S10 Table Kyoto Encyclopedia of Genes and Genomes (KEGG) enrichment analysis of differentially expressed genes. Reference genome: Oryza sativa. -, no significant enrichment; FDR, false discovery rate; D8_AA, samples under drought collected 8 days after anthesis of NILs carrying the non-exotic allele; D8_BB, samples under drought collected 8 days after anthesis of NILs carrying the exotic allele; D11_AA, samples under drought collected 11 days after anthesis of NILs carrying the non-exotic allele; D11_BB, samples under drought collected 11 days after anthesis of NILs carrying the exotic allele; DH11_AA, samples under combined drought and heat collected 11 days after anthesis of NILs carrying the non-exotic allele; D11_BB, samples under combined drought and heat treatment collected 11 days after anthesis of NILs carrying the exotic allele.
(XLSX)
