# Supplementary figures and images for: Transcripts of wheat at a target locus on chromosome 6B associated with increased yield, leaf mass and chlorophyll index under combined drought and heat stress
Source: PLoS One. 2020 Nov 9;15(11):e0241966. doi: 10.1371/journal.pone.0241966 (PMC7652265; doi:10.1371/journal.pone.0241966)

**
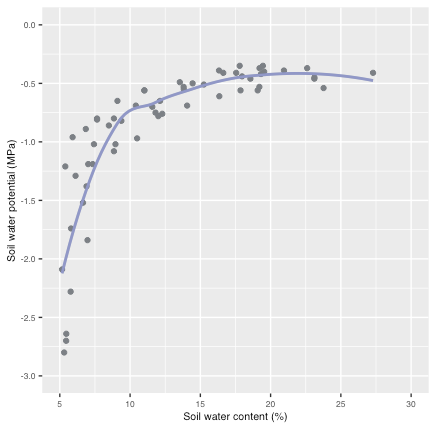
**

**S1 Fig.**

Supplement: S1 Fig — Eight pots of the same size and filled with the same substrate mix as used in the experiment were first watered and then drained until reaching ~5% soil water content. The water content and water potential of the soil were measured daily by coring a of sample of 15 mm diameter and 200 mm length. The water content in soil was determined by calculating the weight difference between fresh soil sample and the oven-dried soil sample (at 65 oC for 72 hours), divided by the oven-dried sample. The water potential of the fresh soil sample was measured using a water potential meter (WP4C, Meter Group, United States) in continuous mode until the value maintained stable. Dots, raw values of the eight pots. Blue line, logarithmic trendline. (DOCX) [file pone.0241966.s001.docx]
